# Cyclotron production of manganese-52: a promising avenue for multimodal PET/MRI imaging

**DOI:** 10.1186/s41181-024-00288-6

**Published:** 2024-08-02

**Authors:** Francesca Porto, Sara Cisternino, Emiliano Cazzola, Giorgia Speltri, Liliana Mou, Alessandra Boschi, Lorenza Marvelli, Giovanni Di Domenico, Antonella Pagnoni, Lucia De Dominicis, Irene Calliari, Claudio Gennari, Licia Uccelli, Gaia Pupillo, Giancarlo Gorgoni, Juan Esposito, Petra Martini

**Affiliations:** 1 Department of Translational Medicine, University of Ferrara, Via Fossato di Mortara, 70 c/o viale Eliporto, 44121 Ferrara, Italy; 2 Legnaro National Laboratories (LNL-INFN), National Institute for Nuclear Physics, Viale dell’Università, 2, 35020 Legnaro, PD Italy; 3 IRCCS Sacro Cuore Don Calabria Hospital, Viale Luigi Rizzardi, 4, 37024 Negrar di Valpolicella, VR Italy; 4 Department of Chemical, Pharmaceutical and Agricultural Sciences, University of Ferrara, Via L. Borsari, 46, 44121 Ferrara, Italy; 5 Department of Physics and Earth Sciences, University of Ferrara, Via Saragat, 1, 44122 Ferrara, Italy; 6 Department of Physics and Astronomy, University of Padova, Via Marzolo, 8, 35131 Padua, Italy; 7 Department of Industrial Engineering, University of Padova, Via Gradenigo, 6/a, 35131 Padua, Italy; 8 Department of Environmental and Prevention Sciences, University of Ferrara, Via L. Borsari, 46, 44121 Ferrara, Italy

**Keywords:** PET/MRI, Manganese-52, Cyclotron production, Spark plasma sintering, Ion exchange chromatography, Mn-radiopharmaceuticals, PET-imaging, Mn-based contrast agents

## Abstract

**Background:**

The integration of positron emission tomography (PET) and magnetic resonance imaging (MRI) holds promise for advancing diagnostic imaging capabilities. The METRICS project aims to develop cyclotron-driven production of ^52^Mn for PET/MRI imaging.

**Results:**

Using the ^52^Cr(p,n)^52^Mn reaction, we designed chromium metal targets via Spark Plasma Sintering and developed a separation procedure for isolating ^52^Mn. Labeling tests were conducted with traditional chelators (i.e. S-2-(4-Isothiocyanatobenzyl)-1,4,7,10-tetraazacyclododecane tetraacetic acid) and the 1.4-dioxa-8-azaspiro[4.5]decane-8- carbodithioate ligand to produce radioactive complexes suitable for PET/MRI applications. Our methodology yielded high-quality ^52^Mn suitable for PET radiopharmaceuticals and PET/MRI imaging. Preliminary studies on phantom imaging using microPET and clinical MRI demonstrated the efficacy of our approach.

**Conclusions:**

The developed technology offers a promising avenue for producing ^52^Mn and enhancing PET/MRI imaging capabilities. Further in vivo investigations are warranted to evaluate the potential advantages of this hybrid imaging technique.

## Background

Introduced in 1990, PET/MRI is a very powerful imaging technique in many clinical and preclinical applications, since it combines MRI (Magnetic Resonance Imaging) morphological sequences with functional PET (Positron Emission Tomography) information in a single scan (Pichler et al. 2008, Boss et al. 2010; Martí-Bonmatí et al. 2010; Musafargani et al. 2018). Currently, multimodal PET/MRI imaging relies on the use of two different chemical compounds, a radiopharmaceutical and a contrast agent (CA). In general, PET images are obtained by administering [^18^F]-based radiopharmaceuticals while magnetic resonance images are acquired using gadolinium-based contrast agents (Pagano et al. 2016). As a result, since the molecules administered to the patient are chemically diverse, they interact differently within the body, and the obtained images are not perfectly overlapping from a diagnostic standpoint. To achieve seamless integration between the two diagnostic techniques, PET and MRI, the research is focusing on developing new compounds having the same chemical structure while keeping both paramagnetic or radioactive properties. If used in combination, these compounds would enable an unprecedented PET/MRI multimodal hybrid imaging, providing enhanced diagnostic capability (Martí-Bonmatí et al. 2010). Manganese appears to be the ideal candidate for this purpose, owing to its paramagnetic properties in the Mn(II) state (d^5^) and the availability of radioactive manganese isotopes (e.g., ^52^Mn) with suitable decay properties for obtaining PET diagnostic images. This allows for the development of radiopharmaceuticals for PET imaging and analogue paramagnetic molecules for MRI applications (Brandt et al. 2019; Lewis et al. 2015; Drahoš et al. 2012; Pan et al. 2011).

Manganese-52 (^52^Mn) decays into the stable isotope chromium-52 (^52^Cr) through the emission of positrons (β^+^) and γ photons with a half-life of 5.591 days (β^+^ = 29.4%, E_ave_β^+^ = 0.24 MeV). Due to its decay characteristics, ^52^Mn offers a promising alternative for the development of new radiopharmaceuticals for diagnostic imaging in PET. In particular, the long half-life of over 5 days allows for its combination with monoclonal antibodies for the development of immunoPET radiopharmaceuticals.

^52^Mn can be produced by cyclotron irradiation of natural chromium (Cr) foils, through the reaction ^nat^Cr(p,n)^52^Mn, however, several impurities may be co-produced as reported in literature (Barrett et al. 2019; El Sayed et al. 2019; Pyles et al. 2023). As a solution, the use of highly ^52^Cr-enriched targets, through the reaction ^52^Cr(p,n)^52^Mn at proton beam energies lower than 16 MeV, turns out to yield ^52^Mn at radionuclide purities as high as requested (Chaple and Lapi 2018; El Sayed et al. 2019; Vaudon et al. 2018).

In this regard the research project METRICS, "*Multimodal pET/mRI Imaging with Cyclotron-produced *^*51/52*^*Mn (β*^+^*emitter/paramagnetic) iSotopes*", funded by the CSN5-INFN (National Scientific Committee 5), aimed to: i) develop the technology needed to get a cyclotron-driven ^52^Mn production for the preparation of radiopharmaceuticals usable for positron emission tomography (PET), and/or for PET/MRI multimodal imaging studies; ii) study and develop Mn-based inorganic coordination complexes (both contrast and radioactive agents) suitable for PET/MRI applications with the final goal to achieve a perfect molecular matching between PET and MRI diagnostic techniques.

So far, the development of Mn-based contrast agents is limited. Despite manganese being a biogenic element, the dose required for contrast administration can have significant consequences, attributed to the release of the Mn(II) ion and its toxicity in vivo at high concentrations (Cersosimo and Koller 2006; Crossgrove and Zheng 2004). One possible strategy to reduce this toxicity is the coordination of the manganese ion with chelating agents that form thermodynamically and kinetically stable complexes. Within the METRICS project, in a previous work we investigated a new class of Mn(II)-dithiocarbamates complexes (Martini et al. 2021; Sguizzato et al. 2022). Particularly, we found that the compound [Mn(II)(DASD)_2_] × 2H_2_O (DASD = 1.4-dioxa-8-azaspiro[4.5]decane-8- carbodithioate), hereafter called Mn-DASD, has excellent contrast properties in water using clinical magnetic resonance (Reale et al. 2023).

Concerning the target material for the production of pure ^52^Mn through the reaction ^52^Cr(p,n)^52^Mn, although ^52^Cr isotopic natural abundance is 83.789%, enriched chromium in the isotope ^52^Cr is a relatively expensive material. Therefore, the choice of a target manufacturing technique that avoids material loss in the process plays a crucial role. In addition, the use of the costly enriched ^52^Cr material would require a method for recovery the Cr waste from the purification, with the possibility of recycling the ^52^Cr material to produce reusable target material and lower the cost of using the enriched ^52^Cr. Foil of enriched ^52^Cr does not exist with convenient dimensions allowing direct irradiation to obtain ^52^Mn. To date, ^nat^Cr targets made by electrodepositions (Pyles et al. 2021) or conventional sintering (Vaudon et al. 2018) are under investigation. However, the electrodeposition of refractory metals like Cr is difficult and the thickness homogeneity is not guaranteed. On the other hand, the conventional sintering allows to obtain almost suitable pellets without backing, however the irradiation tests are not reported.

As we recently described (Cisternino et al. 2022a; b; Esposito et al. 2018; Pupillo et al. 2024), the Spark Plasma Sintering (SPS) technique (also known as Field Assisted Sintering) could be considered a performing and versatile technique for manufacturing robust target that is (i) compatible with high current irradiations, (ii) easy handling in a hot-cell and (iii) compatible with the dissolution reactor, composed by a bottom-opened vial, which allows for selectively dissolution of the target material (Sciacca et al. 2021). In this work, deeper results and analysis on the ^nat^Cr and ^52^Cr targets manufactured be SPS are reported to describe the whole ^52^Mn production chain up to the radiolabeling and imaging with the radioisotope produced. Briefly, the SPS technique is a fast and practical manufacturing method because it allows the sintering of pellets of tailored size, starting from metal or oxide powders, which is the form in which the isotopically enriched materials are usually supplied. Once the desired exact amount of powder is placed in the graphite press, the process takes place in few minutes because pressure and temperature are simultaneously applied. The latter is achieved by the Joule effect due to the electric resistance of the high-intensity current passing through the sample (Anselmi-Tamburini and Groza 2017). The high efficiency of this technique makes it quite advantageous in the case of expensive starting materials, i.e. ^52^Cr. Furthermore, a tight bond between different materials without the need of a filler can be achieved (Hu et al. 2020) leading to a target system composed by the target material attached to a selected backing material. The backing is chosen based on its thermal conductivity and its chemical inertness in the solution used to dissolve the target material after the irradiation to avoid contamination.

Following the target irradiation, a radiochemical separation to isolate manganese from chromium target and contaminants should be applied. Different procedure have been previously described in literature. Barrett et al. (2019) explored a radiochemical isolation method for the production of ^52^Mn from natural chromium targets based on selective Cr(OH) precipitation with 5 M NH_4_OH in the presence of 5 M NH_4_Cl at pH∼8 from the Mn-rich target solution achieving a 85 ± 2.6% radiochemical yield.

Pyles et al. (2021) and later Pyles et al. (2023) described the use of a two- or three-column radiochemical protocol based on anion exchange resin and hydroalcoholic mobile phase to separate manganese from chromium for the production of ^52^Mn, highlighting the effectiveness of an automatic process to improve safety and production efficiency, achieving recovery yields up to 94.5 ± 2.2% in 8.2 ± 0.6 h of processing (Pyles et al. 2021; Pyles et al. 2023).

In this work we reported the developed technology to yield ^52^Mn by medical cyclotron via the ^52^Cr(p,n)^52^Mn nuclear reaction route including: (i) the design and production of chromium metal targets to fit the size of the solid target station of a medical cyclotron using the Spark Plasma Sintering (SPS) technique; (ii) the development of an automated and efficient procedure for separating ^52^Mn from the Cr bulk using a cassette-based automatic module with a solid target dissolution system. Finally, labeling tests with the ligand DASD and with the S-2-(4-Isothiocyanatobenzyl)-1,4,7,10-tetraazacyclododecane tetraacetic acid (DOTA-SCN) ligand were conducted. The ^52^Mn-DOTA-SCN complex was exploited to carry out preliminary studies on phantom, using a microPET imaging system. Moreover we recently performed phantom imaging with a clinical MRI on the non-radioactive complex analogue Mn-DASD, aimed assessing the contrast enhancement compared to a gadolinium contrast agent gold standard, as recently described by Reale et al. (Reale et al. 2023).

## Results

### Target manufacturing

Pellets of both natural chromium and ^52^Cr-enriched have been produced and bonded to Au/Nb backing materials using the SPS technique.

**Fig. 1:**
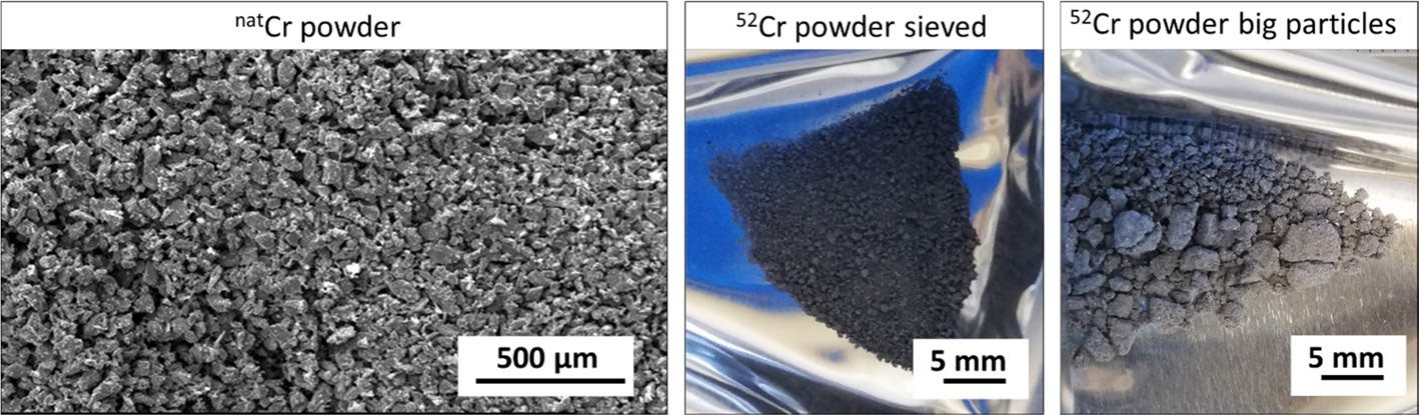
SEM image of ^nat^Cr powder (left) and picture of the ^52^Cr fine sived powder (middle), and big powder particles (right)

In Fig. 1 the SEM (Scanning Electron Microscopy) image of the ^nat^Cr powder shows its irregular shape (not spherical) and its size less than 100 µm. Instead, ^52^Cr powderl has irregular shapes and a wide size range as can be seen in Fig. 1.

**Fig. 2:**
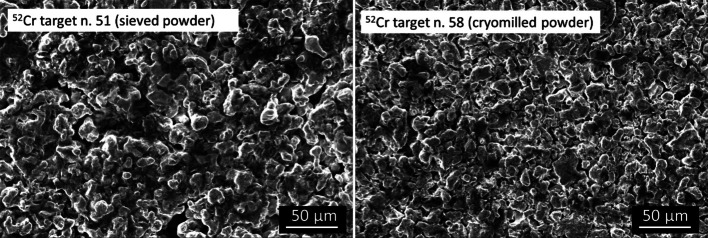
SEM images of the ^52^Cr targets realized with sieved powder (left) and cryomilled powder (right)

The results of the sintering process to obtain Cr pellet from powder are summarized in Table 1. The mean and standard deviation of the thickness and relative density of the ^nat^Cr pellet obtained using 950 °C (heating rate 200 °C/min) and 81 MPa, was 433.8 ± 52.9 µm and 63.6 ± 2.9%, respectively (n = 11). As expected, the ^52^Cr pellet realized using the sieved powder has a relative density lower than the pellet coming from the cryomilled powder, 51.3 ± 2.9% (n = 2) and 60.4 ± 0.5% (n = 2), respectively. The differences of the powder size and the density can be observed from the surface SEM images of the pellets obtained from sieved and cryomilled powder, as well (Fig. 2). Only superficial oxidation and carbon contamination were detected from the EDS (Energy Dispersive x-ray Spectroscopy) analysis of the ^52^Cr targets.

**Table 1:** Results of ^nat^Cr and ^52^Cr pellet sintering

Tot. pellets	Material	Mass [mg]	Thickness [µm]	Relative density %
11	^nat^Cr	163.5 ± 8.7	433.8 ± 52.8	63.6 ± 2.9
2	^52^Cr-sieved powder	162.5 ± 0.7	551.5 ± 49	51.2 ± 2.9
2	^52^Cr cryomilled powder	164.0 ± 1.4	463.3 ± 10.5	60.4 ± 0.5

The relative density was calculated with reference to the theoretical one ρ_th_ = 7.19 g/cm^3^. Mean and st.deviation of the the mass, thickness and relative density are reported over the total number of pellets realized

The completed targets composed by Cr pellet attached to Au/Nb disc had excellent solidity; indeed, not even the use of a scalpel or cutter enables them to be detached.

**Fig. 3:**
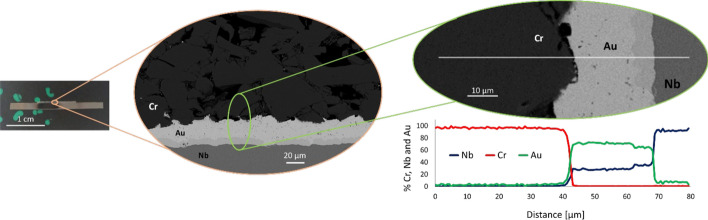
Metallurgical interphase analysis of the target Cr-Au/Nb at different magnitudes: picture and SEM images of the cross-section view. The EDS line scanning is also reported

In Fig. 3 the target cut along the diameter and prepared for the metallurgical interphase analysis is shown. The corresponding SEM images at different magnifications acquired by Backscattered Electrons point out the adhesion of the different materials and the EDS analysis along the interphase. From the metallurgical point of view, the analyses indicate that there is no diffusion between Cr and Au as expected from the phase diagram (Okamoto and Massalski 1985a), confirming they are mechanically attached. From the EDS line scanning it seems that the Nb is present in the Au foil, but this is due to the misidentified lines Nb Lα (2.166 keV) and Au Mα (2.120 keV) (Newbury 2009). Indeed, from punctual analysis, the Nb is not present in the Au layer. Only a thin layer (about 5 µm) of intermetallic phase (AuxNbx (Okamoto and Massalski 1985b)) is observed in proximity of the Nb disc.

The efficiency of the material usage with SPS is more than 95% over many experiments (95.4% ± 3.8%, n = 15). Thus, the starting amount of Cr powder used for the realization of a desired pellet (in this work the initial mass was 163.4 ± 7.4 mg, n = 15) is fruitful for a pellet of 156.4 ± 10.5 mg, corresponding to a loss of 7.4 ± 5.9 mg for each experiment.

### Cr/Mn separation test

**Fig. 4:**
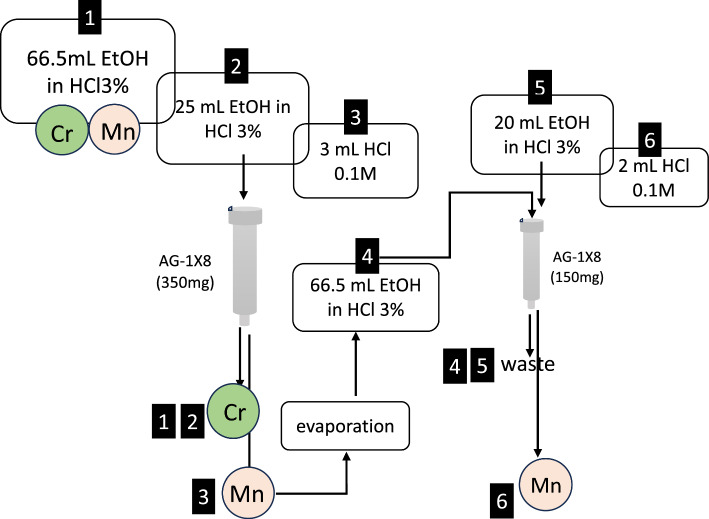
Scheme of the Cr/Mn separation procedure based on the use of two AG1-X8 anionic resin. **1**.Loading a 3% HCl solution containing the mixture Cr/Mn on the anionic resin; **2**. Washing with 25 mL of 3% HCl in EtOH; **3**. Mn elution with 3 mL of HCl 0.1 M; Solvent evaporation and dissolution with 2 mL of HCl 37% and 64.5 mL of EtOH; **4**. Loading on the second AG1-X8; **5**. Washing with 20 mL of 3% HCl in EtOH; **6**. Mn elution with 2 mL of HCl 0.1 M

**Fig. 5:**
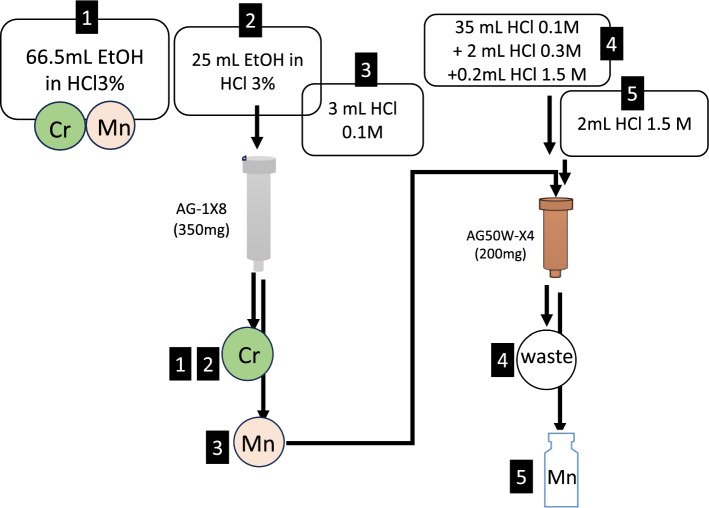
Scheme of the Cr/Mn separation procedure combining an AG1X-8 resin with an AG50W-X4 resin. **1**. Loading a 3% HCl solution containing the mixture Cr/Mn on the anionic resin; **2**. Washing with 25 mL of 3% HCl in EtOH; **3**. Mn elution with 3 mL of HCl 0.1 M and loading on the AG50W-X4 resin; **4**. Washing with 35 mL HCl 0.1 M, 2 mL HCl 0.3 M and 0.2 mL HCl 1.5 M; **5**. Mn elution with 2 mL of HCl 1.5 M

Two different separation procedure based on ionic-exchange chromatography have been developed to separate Mn from the chromium target. The first procedure replicates a known method involving double anion exchange resin (Fig. 4), where Cr is eluted using a hydroalcoholic solution and Mn with 0.1 M HCl (Graves et al. 2015; Pyles et al. 2023). The second method utilizes a series arrangement of first anion and second cation exchange resins (Fig. 5). Here, manganese eluted from the first resin is directly transferred to the second resin, eliminating time-consuming evaporation steps, thus reducing processing time and waste volume compared to the first procedure. The results of bench experiments conducted with non-irradiated target, spotted with a manganese standard to simulate cyclotron production conditions, and following the double anion exchange resin procedure outlined in Fig. 4, confirmed the effectiveness of the published separation methods. This method enabled a manganese recovery yield of 88 ± 5% (n = 4) in over four hours. The chromium content in the manganese eluate after passing through the first anion exchange resin is approximately 0.7 mg. Subsequently, after passing through the second anionic resin, the chromium content in the final product is significantly reduced to parts per billion levels (below the detectable limit of the instrument: Cr = 11.9 ppb).The use of a cationic resin as the second resin dramatically reduces the procedure time to just a couple of hours, with a chromium content in the manganese eluate < 10 ppm and a manganese recovery yield at 85 ± 9% (n = 4). Figure 5 outlines the optimized procedure, which involves combining an AG1X-8 resin with an AG50W-X4 resin. This procedure was therefore automated and used for the radiochemical processing of the irradiated chromium target for the cyclotron-production of ^52^Mn.

### Target irradiation and processing

No damages of the targets were observed after the irradiation tests under the proton cyclotron beam (ACSI TR19/300 cyclotron), at increasing currents, as demonstrated by the picture in Table 2, where an example of ^nat^Cr target and ^52^Cr target before and after the irradiation is shown. The irradiated ^nat^Cr targets were also used for the optimization of the radiochemistry process. Finally, the enriched ^52^Cr target was irradiated at 14.2 µA, 16.8 MeV for 45 min to produce ^52^Mn used for the labeling and imaging tests. The total activity of ^52^Mn produced on the enriched target at the End Of Bombardment (EOB) was 127 MBq.

**Table 2:** Pictures of one ^nat^Cr target and ^52^Cr target before and after the irradiation using the reported parameters

Target type	Photo as prepared	Photo after irradiation	Energy [MeV]	Current [µA]	Time [min]
^nat^Cr target			16.8	10–50	20–30
^52^Cr target			16.8	14.2	45

In the picture the ^nat^Cr target after the irradiation placed in the reactor for dissolution process is shown

**Fig. 6:**
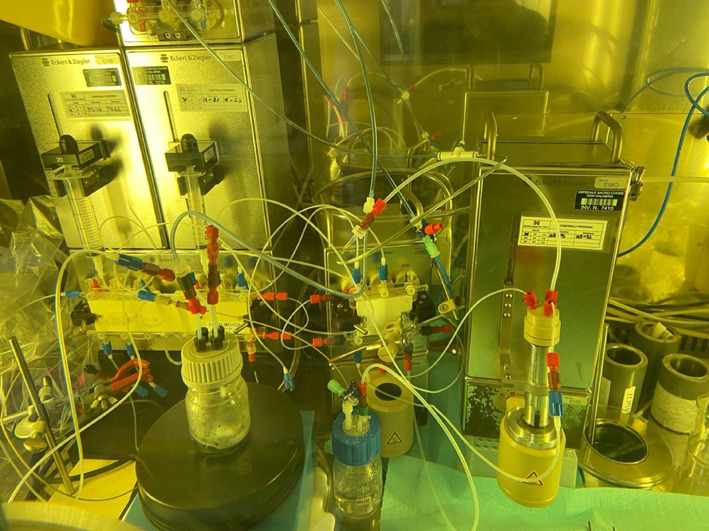
Automatic module assembly by using Eckert & Ziegler cassette-based modular units, on the left the dissolution reactor especially realized to fit the target as described by Sciacca et al. 2021

The automated target processing protocol (Fig. 6) based on anion and cation exchange resins was applied to both natural and enriched chromium targets for manganese recovery. The preliminary tests (n = 4) involving ^nat^Cr target allowed to recover 75 ± 5% of manganese in 4 h of processing (1 h for target dissolution, 1 h for evaporation and 2 h of columns loading, washing and elution). The radiochemical processing of the enriched ^52^Cr target resulted in a manganese recovery yield of approximately 78%.

The gamma spectrometry analysis revealed the presence of chromium and cobalt isotopes in the load and wash samples, while no contamination was detected in the final ^52^Mn product.

**Fig. 7:**
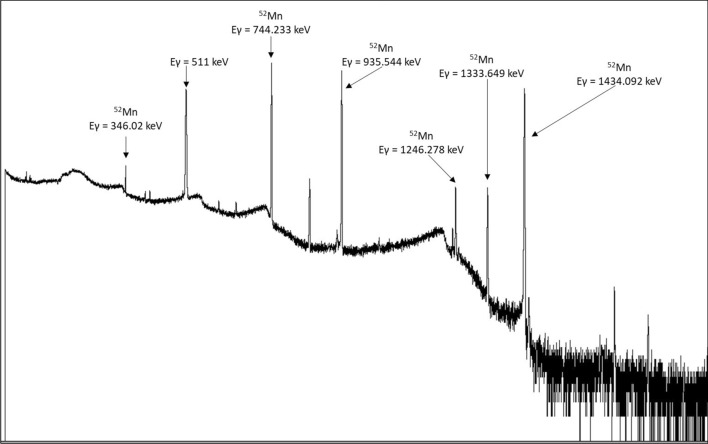
Image of the HPGE γ-spectra of the product at EOP

In Fig. 7, as an example, a spectrum of the ^52^Mn product sample obtained at the End Of the radiochemical Purification (EOP) procedure is shown, where, in addition to the background peaks, only those relating to ^52^Mn are present.

### Radiolabeling and PET imaging

**Fig. 8:**
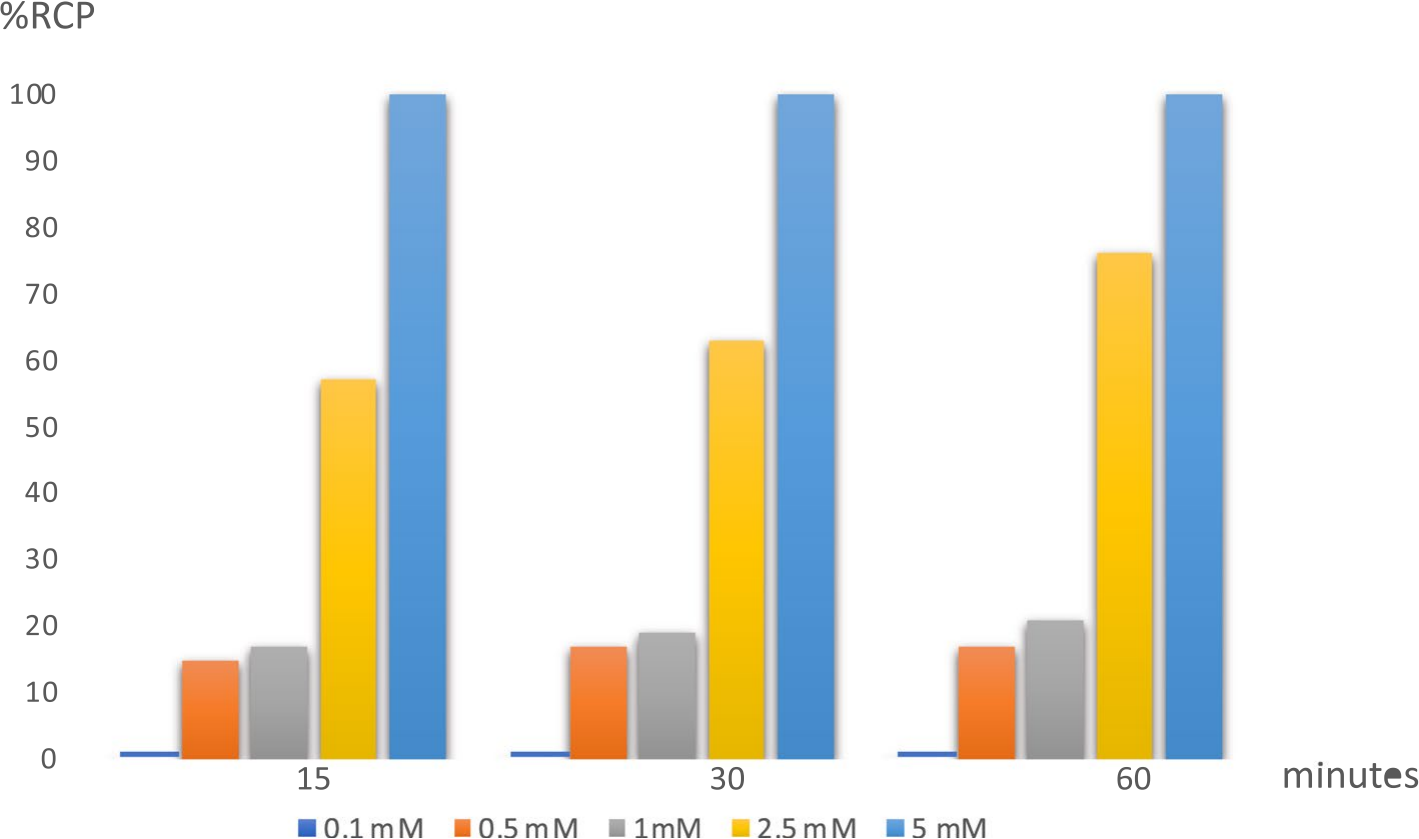
Radiochemical purity (%RCP) of the [^52^Mn]Mn-DOTA-SCN compound obtained incubating [^52^Mn]MnCl_2_ with different concentrations of the ligand DOTA-SCN, and at different time

The ligand DOTA-SCN was successfully labeled with the purified [^52^Mn]MnCl_2_, at pH 5.5 with a radiochemical purity exceeding 99% reached in 15 min at 60 °C. In Fig. 8 the percentage of the radiochemical purity of the [^52^Mn]Mn-DOTA-SCN compound, obtained incubating [^52^Mn]MnCl_2_ with different concentrations of the DOTA-SCN ligand and incubation times, is reported.

The ligand DASD was labeled by incubating aliquots of purified ^52^Mn with 200 μg of ligand (DASD) in the presence of carbonate buffer at pH 9. The radiochemical purity determined by TLC (Thin Layer Chromatography) was 95%.

**Fig. 9:**
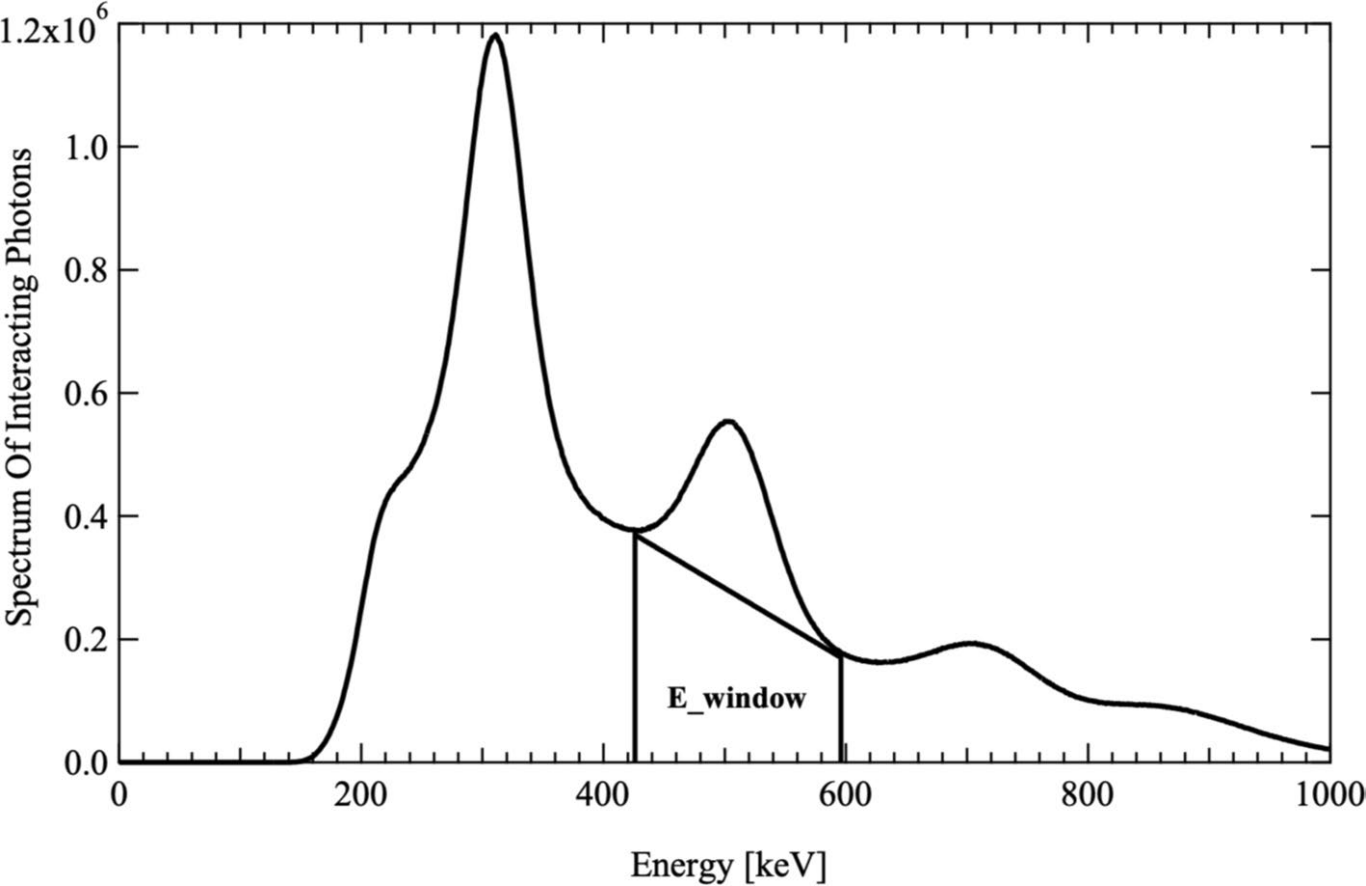
Spectrum of interacting photons into the PET detector. The two vertical lines indicate the energy window used to reconstruct the PET data, while the blue line separates the region below the peak of the 511 keV corresponding to the photons scattered by the region of the non-scattered photons

**Fig. 10:**
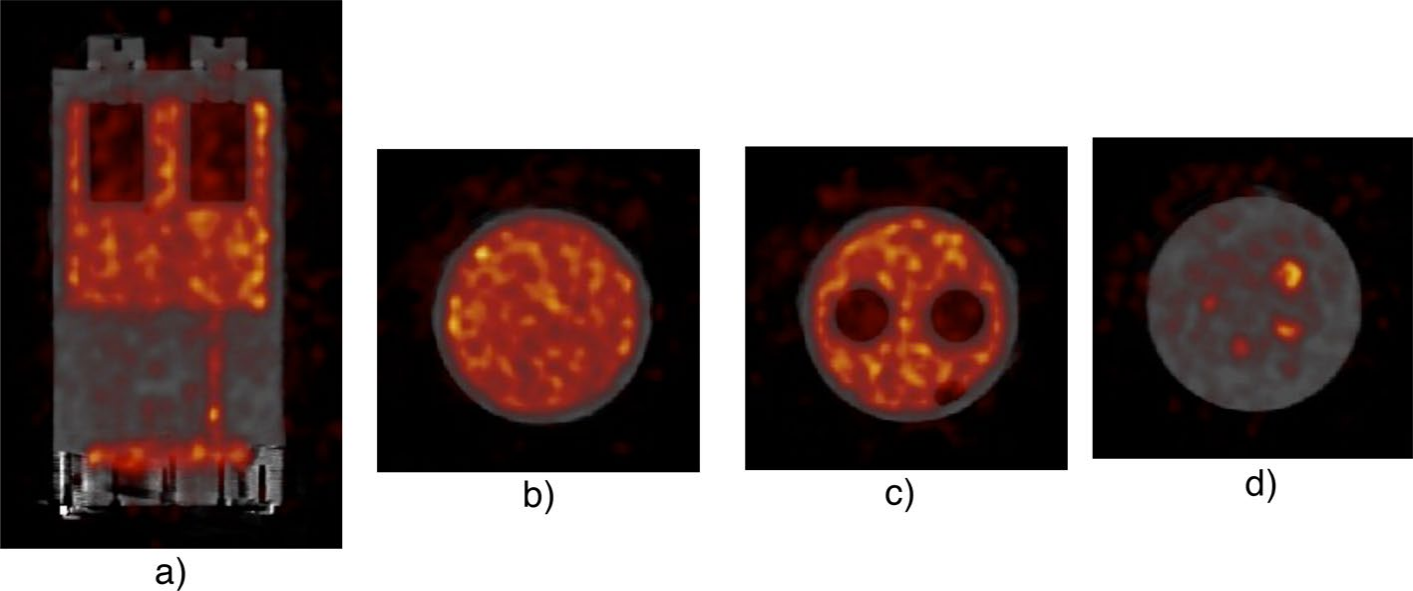
Reconstructed images form NEMA NU-4 image quality phantom. PET data was reconstructed with attenuation and scatter correction by using the iterative method: **a** a single sagittal slice is shown highlighting the different sections in the phantom; **b** a single transverse slice through the uniform section of phantom; **c** a single transverse slice through the section of the phantom containing air; and **d** a single transverse slice through the 5 rods region

Tomographic data of the NEMA NU-4 Image Quality phantom on the MOLECUBES PET/CT scanner are reported in Fig. 9. PET images were reconstructed, image uniformity, contrast recovery, and accuracy of data corrections were calculated. The results obtained for the ^52^Mn PET images are reported in Table 3 and Fig. 10.

**Table 3:** Results of image uniformity, contrast recovery, and accuracy of data

Uniformity	Accuracy of corrections	Recovery Coefficient (RC)
15%	33% ± 13%

## Discussion

### Target manufacturing

The Spark Plasma Sintering (SPS) technique, using the tailored version of SPS machine, TT_Sinter, was successfully adopted for manufacturing ^52^Cr target composed by Cr pellet bonded to a support material composed by Nb disc and thin Au layer.

The possibility to sinter Cr pellet starting from the exact amount of powder material needed and the adhesion between the target material and backing allowed to realize a target system that fulfills the main requirements for a suitable cyclotron target (i.e., no losses of the expensive material, a uniform and optimized layer thickness; a high chemical purity level; a high heat transfer effectiveness; good mechanical properties; and easy handling).

The first tests were performed using ^nat^Cr powder and it was observed that the powder size in the range of 40–100 µm can be uniformly distributed inside the graphite press in order to obtain uniform pellet thickness and a density of about 63%. Because of the shape and the size of the ^52^Cr material supplied by the provider, (Fig. 1) a preliminary powder preparation was necessary to reduce the granulometry size from 3 mm up to at least 100 μm. Therefore, part of ^52^Cr powder was sieved in order to select the smallest powder and the remaining ^52^Cr powder larger-size particles were cryomilled to reduce the granulometry as suggested by Cisternino et al. (Cisternino et al. 2023). The irradiation tests and the production of ^52^Mn using targets made from cryomilled ^52^Cr powder are not described in this work, they will be the subject of future studies. SEM analysis of enriched ^52^Cr powder was not performed to avoid the lost of the expensive powder.

The SPS parameters used for the realization of ^nat^Cr allowed to realize ^52^Cr pellets with relative densities of about 50% and 60% based on the powder size used. In this conditions, the powder size influences the obtained pellet density, smallest the powder higher the density. However, the pellet porosity could be an advantage for the adhesion with the support disk (mechanical adhesion is enhanced) and for the chemical reactivity (in the radiochemistry step).

In the previous work by the authors (Sciacca et al. 2021) the targets were composed of Cr pellet attached to Au/Cu disc, instead, in this work, the Cu backing was substituted by Nb backing to avoid the high activation after the irradiation and make safer the operations. From the metallurgical point of view (Fig. 3), a good adhesion between the materials (Cr-Au-Nb) is achieved, guaranteeing an efficient heat dissipation during the irradiation, despite the lower thermal conductivity of Nb (53.7 W/m·K) respect to Cu (395 W/m·K). Indeed, no damage of the targets after irradiation at 50 µA and 16.8 MeV was observed (Table 2), demonstrating the target thermo-mechanical stability up to 1 kW/cm^2^ thermal power density. Therefore, the feasibility of Cr targets by the SPS technique overcomes the drawbacks of the other explored techniques, such as ^nat^Cr foil, powder pressing, use of Cr_2_O_3_ or electrodeposition (Kretowicz et al. 2023; Pyles et al. 2023). This latter manufacturing method allows the enriched powder material to be recycles, as described by Kretowicz et al. (Kretowicz et al. 2023), however, the adhesion of electroplated Cr to the backing material and the increase of the thickness and thickness uniformity should be improved to produce a higher ^52^Mn yield.

As already explained, one advantage of using SPS technique is its efficiency in terms of material usage, more than 95% of the material employed for one process is useful for the target, thus avoiding significant losses of precious material, i.e.^52^Cr. However, in this work a material recycling process was not studied.

25 μm Au layer, inserted between Cr pellet and Nb disc, is sufficient to keep its protective aim for the dissolution step. Indeed, in the γ-spectrum of an aliquot of the dissolved Cr, only the energy peaks corresponding to manganese and chromium isotopes were identifiable (Fig. 7). No radioactive contaminants coming from the backing material were detected.

### Cr/Mn separation test and irradiated target processing

The ion exchange chromatography method has been chosen as the most efficient for automating two procedures of Cr/Mn separation. The first procedure replicates a known method involving double anion exchange resin (Fig. 4) (Graves et al. 2015; Pyles et al. 2023), the second method utilizes a series arrangement of first anion and second cation exchange resins (Fig. 5).

While the separation method involving a double anion exchange resin proves to be highly efficient, it resulted in being very slow due to time-consuming evaporation steps and sensitive to loading flow rate. For an efficient separation and recovery of Mn the flow rate should be below 1 mL/min, better at 0.5 mL/min of over 60 mL of solution for each of the two anionic resins, thus resulting in about 2 h of loading time. Moreover, two time-consuming evaporation steps are necessary to reconstitute the sample in HCl 3% solution in EtOH before each loading onto each anionic resin. These conditions significantly prolong the processing time (exceeding four hours) and result in the generation of large volumes of radioactive waste. In contrast, utilizing a cationic resin as the second resin dramatically reduces the procedure time to just a couple of hours, since Mn eluted from the first anionic resing is already in suitable conditions to be loaded and retained on the cation exchange resin while maintaining the chromium content in the final Mn-rich product at the parts per million (ppm) level. The second method was thus selected for automation and involved in the separation and purification of Mn from irradiated natural and enriched Cr targets. The automated procedure resulted in an efficient recovery yield (75 ± 5%) in 4 h. The product was analyzed through gamma spectrometry, revealing that no other radionuclides were present other than ^52^Mn. Comparing these results with the literature, which reports a recovery yield of up to 94.5 ± 2.2% and a processing time of 8.2 ± 0.6 h (Pyles et al. 2021, 2023), we can conclude that the recovery yield of the procedure described here is improvable, but this process significantly gains in the processing time, which is halved.

### Radiolabeling and PET imaging

The chemical purity of the ^52^Mn obtained was further assessed through a preliminary chelation test with the gold standard of the chelators, DOTA.

**Fig. 11:**
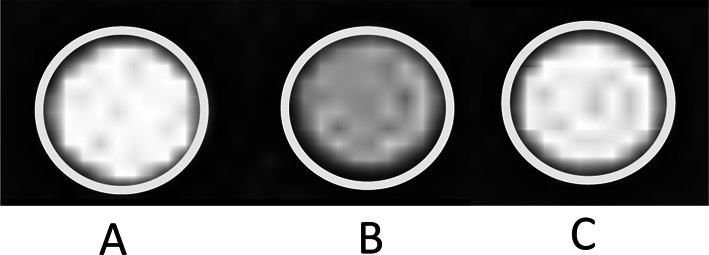
T1-weighted MR images of glass tubes containing Gadobenato dimeglumina (**A**), Mn(II)Cl_2_ × 4H_2_O (**B**), [Mn(II)(DASD)_2_] × 2H_2_O (**C**) in water at concentrations 3 mM obtained with 1.5 T a clinical Siemens Magnetom Aera (Reale et al. 2023). Original picture

Moreover, we recently reported the development of a mononuclear compound [Mn(II)(DASD)_2_] × 2H_2_O (Mn-DASD) (DASD = 1.4-dioxa-8-azaspiro[4.5]decane-8- carbodithioate anion) potentially usable as a contrast agent for magnetic resonance imaging. Studies investigating the properties of paramagnetic imaging in water using a clinical magnetic resonance system revealed that the contrast generated by the complex Mn-DASD is comparable to that produced by gadolinium complexes currently employed in medicine as paramagnetic contrast agents. In Fig. 11 are reported, as an example, the T1-weighted MR images of glass tubes containing a Gd-based contrast agent, Mn(II)Cl_2_ × 4H_2_O and the Mn-DASD compound collected with a clinical Magnetic Resonance (MR) (Reale et al. 2023).

Therefore, to produce ^52^Mn-radiopharmaceuticals and analogous paramagnetic complexes of manganese for PET/MRI multimodal diagnostics applications, the preparation of the analogous compound with cyclotron-produced ^52^Mn has been carried out. The chromatographic comparison on TLC appears to confirm the identity of the product, and further characterization tests are foreseen.

The PET image quality evaluation using the ^52^Mn was assessed by acquiring tomographic data of the NEMA NU-4 Image Quality phantom on the MOLECUBES PET/CT scanner. A scatter correction is needed due to the huge scatter contribution of high energy photons emitted in ^52^Mn decay as shown in Fig. 9, containing the spectrum of photons interacting in PET detector system. The images collected on phantom with a preclinical scanner tomograph confirm the quality of the product.

## Conclusions

In conclusion, the activities carried out within the METRICS project aimed to develop cyclotron-driven production of ^52^Mn for PET/MRI imaging studies have been summarized. The project focused on two main objectives: advancing the technology for ^52^Mn production and studying Mn-based inorganic coordination complexes suitable for PET/MRI applications.

Specifically, we reported the development of technology to produce ^52^Mn via the ^52^Cr(p,n)^52^Mn reaction, including the design and production of chromium metal targets using the SPS technique and the development of an efficient separation procedure for isolating ^52^Mn from the chromium bulk. Additionally, labeling tests were conducted using traditional chelators like DOTA and the DASD ligand to produce radioactive complexes and analogous to non-radioactive contrast agents developed for MRI studies.

The produced ^52^Mn-DOTA-SCN complex underwent preliminary studies on phantom imaging using a microPET imaging system, while non-radioactive complex Mn-DASD was tested on clinical MR to verify contrast enhancement compared to a gadolinium contrast agent gold standard. Results indicated the effectiveness of the developed technology in producing high-quality ^52^Mn suitable for PET radiopharmaceutical preparation and PET/MRI applications in combination with previously developed non-radioactive analogs. Further in vivo studies combining the radiopharmaceutical and contrast agent are warranted to assess the advantages of this unprecedented hybrid technique.

## Methods

### Target manufacturing

The TT_Sinter machine developed by the University of Pavia and INFN Pavia in collaboration with LARAMED group was used to produce ^nat^Cr and ^52^Cr targets (Cisternino et al. 2022a, b; Esposito et al. 2018; Pupillo et al. 2024).

^nat^Cr powder (≥ 99% trace metal basis, -325 mesh) was purchased by Sigma Aldrich (St. Louis, Missouri, USA). Isotope-enriched ^52^Cr material (enrichment level 98.859 ± 0.008%) purchased by ISOFLEX (San Francisco, California, USA) was used for the final target to produce ^52^Mn.

Nb disc (Ø23.5 mm, thickness 1.6 mm, purity 99.99% purchased by Goodfellow, Huntingdon, England) covered with thin Au layer (purity 99.95%, Alfa Aesar—Massachusetts, USA—or Goodfellow Ø20 mm, thickness 25 µm) was used as backing support.

Nb disc covered with a thin Au layer was used as a backing support.

**Fig. 12:**
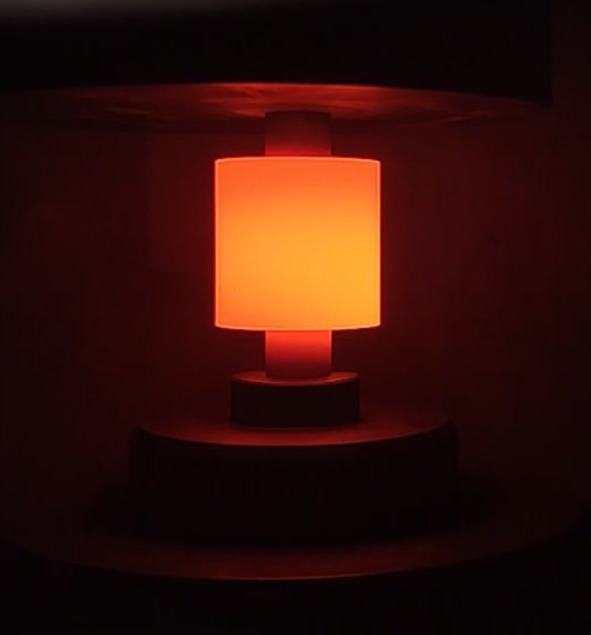
Image of the graphite press in the vacuum chamber during the Cr powder sintering

The SPS procedure and parameters are described in Table 4. It consisted in three steps: (i) Cr pellet preparation starting from powder; (ii) preparation of backing support (i.e., adhesion of Au layer onto Nb disc); (iii) adhesion of Cr pellet on backing. In Fig. 12 a picture of the press during the Cr powder sintering is shown as an example.

**Table 4:** SPS dparameters and procedures used for Cr target manufacturing. Pictures of each step are provided

	Step 1. Cr pellet	Step 2. Au/Nb adhesion	Step 3. Cr–Au/Nb adhesion
Heating rate [°C/min]	200	200	200
Temperature [°C]	950	700	800
Pressure on sample [Mpa]	81	11.25	14
Dwell time [min]	5	3	3
Preparation of the materials in the graphite press			
Results

Cr pellets size was 10 mm diameter, the beam size of the cyclotron, and about 400 µm thickness. The starting Cr powder amount was about 165 mg.

Au layer was attached on Nb to isolate the Cr pellet from the Nb backing and avoid potential contamination in the final solution coming from the backing during the dissolution step.

To center the Cr pellet on the backing, a cave (ca. 300 µm depth), coaxial to the graphite punch, was machined.

Metallurgical interface analysis was performed on ^nat^Cr target to observe the adhesion between the materials. The metallographic sample preparation was performed at metallurgy department of the DII at University of Padova following a rigid step-by-step process, thus avoiding misleading chemical and structural alterations of the sample surface: (i) sectioning with SiC cut-off wheel; (ii) incorporation in acrylic resin (Polyfast) at 180 °C, 20 kN for 7 min; (iii) manual grinding on SiC grinding paper (grit 320–4000) using H_2_O as lubricant; (iv) Polishing cloth with 6–1 µm polycrystalline diamond suspension and finally mirror polishing with 0.2 µm colloidal silica suspension.

SEM–EDS analyses were performed to analyse the ^nat^Cr powder, the surface of the pellets, and the interphase analysis characterization.

The obtained targets were cleaned with ethanol and alpha wipe before irradiation, to eliminate the residual graphite powder on the surfaces.

The target size fits the target station of the TR19 cyclotron at Sacro Cuore Don Calabria Hospital (Negrar di Valpolicella, VR, Italy) where the irradiation and radiochemistry processes were carried out.

### Cr/Mn separation test

Two ion exchange-based purification methods, using two anionic AG1-X8 resins (100–200 mesh, Bio-Rad, California, USA) or one AG1-X8 in series with a cationic AG50W-X4 resin (100–200 mesh, Bio-Rad, California, USA), were assessed through preliminary bench experiments using non-radioactive manganese.

The determinations of Mn and Cr were made with an ICP-OES device (Optima 3100 XL, Perkin-Elmer, Shelton, USA) equipped with an axial torch, segmented array charge-coupled device detector, and low-flow Gem- Cone nebulizer with cyclonic spray chamber for sample introduction and choosing, among the several wavelengths, the readings at 267.716 nm for Cr and 257.610 for Mn. The manganese and chromium concentrations were obtained by comparison with a calibration curve obtained after measuring known concentrations of the metal ion (Visentin et al. 2016).

### Target irradiation and processing

First irradiation tests were performed at increasing proton beam current to assess the thermo-mechanical stability of the chosen target configuration. Several tests at 16.8 MeV at increasing beam currents (10–20-50 µA), for a few (3–5 min) up to 30 min on ^nat^Cr targets and one irradiation at 16.8 MeV, 14.2 µA for 45 min on enriched ^52^Cr target were performed. After irradiation, targets were transferred with the pneumatic transfer system to the hot-cell for visual inspection and radiochemical processing.

The irradiated target was dissolved in concentrated hydrochloric acid (HCl) at 70 °C in an automatic module assembled by using Eckert&Ziegler cassette-based modular units and containing the dissolution reactor especially realized to fit the target and carry out the purification in a single module, as shown in Fig. 6 (Sciacca et al. 2021).

The resulting solution was evaporated to dryness and diluted to 3% HCl in ethanol and then applied to a column packed with AG1-X8 resin. Chromium was eluted using a 3% HCl solution in ethanol. Subsequently, manganese was eluted with 3 mL of 0.1 M HCl and directly loaded onto an AG50W-X4 resin. After purification with HCl at varying concentrations, the isolated ^52^Mn was eluted with 1.5 M HCl (Fig. 5).

The determination of the radiochemical separation efficiency, the total ^52^Mn activity and the Radio Nuclidic Purity (RNP) evaluation were conducted through γ-spectrometry analyses, while the quantification of chromium in the final ^52^Mn solution was accomplished via ICP-OES analysis.

Gamma spectrometric measurements were performed using a High-Purity Germanium (HPGe) detector (Sw GENIE II Canberra, Meriden, CT, USA). The efficiency calibration used in the data analysis was carried out in the energy window 17 keV to 1923.1 keV for three different geometries (Eppendorf 1 ml; vial 5 ml; vial 1 ml) by using a multi-peak certified liquid source (purchased by Eckert & Ziegler and containing the reference radionuclides ^241^Am, ^109^Cd, ^139^Ce, ^57^Co, ^60^Co, ^137^Cs, ^113^Sn, ^85^Sr, ^88^Y, and ^51^Cr), with the Genie 2000 software. Activity measurement was carried out with a Capintec, Inc. dose calibrator, model CRC-25PET, periodically subjected to a quality control program. An appropriate factor was set for each isotope measured. The γ-spectra were analyzed using the JRadView software, developed at the INFN-LNL for nuclear physics experiments.

### Radiolabeling

#### Chelation of purified ^52^Mn with DOTA-SCN

An amount of 100 μL solution containing DOTA-SCN at different concentrations (5 mM, 2.5 mM, 1 mM, 0.5 mM, 0.1 mM) has been prepared in 1 mL vials. Then 50 μL of purified [^52^Mn]MnCl_2_ (555 kBq), buffered with 17 μl of Na_2_CO_3_ (0.5 M) and 150 μL of NaOAc (0.25 M) at pH 5.5, were added to each vial. Vials were then incubated for 60 min at 60 °C. The percentage of chelated ^52^Mn was determined via thin layer chromatography (TLC) using gel silica plates developed in 0.1 M HCl mobile phase. Each spot (2 μL) was performed 15, 30, and 60 min respectively after the start of the incubation of each labeling reaction. The evaluation of the TLC plate was performed with a Cyclone Plus 4312 (PerkinElmer) with phosphor storage system. [^52^Mn]Mn-DOTA-SCN remained at the origin while free ^52^Mn moved with the mobile phase.

#### Chelation of purified ^52^Mn with DASD

Preliminary labeling tests were carried out by incubating [^52^Mn]MnCl_2_ with the sodium salt of the 1.4-dioxa-8-azaspiro[4.5]decane-8- carbodithioate (DASD) ligand. Briefly, the purified [^52^Mn]MnCl_2_ (555 kBq) was dried, resumed with 100 μL of EtOH, and then incubated with 200 μg of DASD, previously dissolved in 500 μL of EtOH. The pH of the solution was then adjusted at 9 by the addition of 100 mL of carbonate buffer 0.5 M. The resulting mixtures were stirred at either room temperature (RT) or 70 °C and analyzed at specified time-points by radio-TLC (stationary phase: SiO_2_, mobile phase: sodium citrate 0.1 M). In this chromatographic condition [^52^Mn]Mn-DASD shows Rf = 0.3 while free ^52^Mn moved with the mobile phase.

#### PET imaging

A preliminary assessment of the imaging quality of ^52^Mn produced from ^52^Cr enriched targets has been carried out on NEMA NU-4 Image Quality phantom with a X-CUBE, and β-CUBE scanners by MOLECUBES for micro-CT and micro-PET tomography (Molecubes, Gent, Belgium). The phantom was filled with 3.6 μCi of ^52^Mn and a 120-min PET scan was acquired with the phantom placed at the center of the scanner. At the end of the PET scan, the imaging bed was transferred to the X-CUBE scanner and a CT scan using the general-purpose acquisition protocol was acquired. The CT image was used for performing PET attenuation and scatter corrections. PET Images were reconstructed using 30 iterations of iterative OSEM. Images were reconstructed into a 96 × 96 transverse matrix with cubic image voxels measuring 0.8 mm. As prescribed by the NU-4 standard, image uniformity, contrast recovery, and accuracy of data corrections were calculated over the different sections of the reconstructed images of the NU-4 image quality phantom. Image uniformity was measured with a 10 mm long volume of interest (VOI) covering the central 75% of the uniform section. The accuracy of PET data corrections was measured over a central 7.5 mm VOI that was half the diameter of the air-filled compartments. Contrast recovery (CRC) was measured over a region of interest (ROI) that is twice the physical diameter of each of the five rods.

## Data Availability

The raw data supporting the conclusions of this article will be made available by the authors on request.

## Abbreviations

PET: Positron emission tomography
MRI: Magnetic resonance imaging
DOTA-SCN: S-2-(4-Isothiocyanatobenzyl)-1,4,7,10-tetraazacyclododecane tetraacetic acid
DASD: 1.4-Dioxa-8-azaspiro[4.5]decane-8- carbodithioate
DBCA: Gadolinium-based compounds
FDA: Food drug administration
SPS: Spark plasma sintering
FAST: Field assisted sintering technique
SEM: Scanning electron microscopy
EDS: Energy dispersive x-ray spectroscopy
ICP-OES: Inductively coupled plasma – optical emission spectroscopy
